# Myocardial Perfusion SPECT Imaging in Patients after Percutaneous Coronary Intervention

**DOI:** 10.2174/157340310791162677

**Published:** 2010-05

**Authors:** Panagiotis Georgoulias, Varvara Valotassiou, Ioannis Tsougos, Nikolaos Demakopoulos

**Affiliations:** 1 Department of Nuclear Medicine, University Hospital of Larissa, Larissa, Greece; 2 Department of Nuclear Medicine, NIMTS Hospital, Athens, Greece

**Keywords:** SPECT, Myocardial Perfusion Imaging (MPI), Percutaneous Coronary Intervention (PCI), Revascularization.

## Abstract

Coronary artery disease (CAD) is the most prevalent form of cardiovascular disease affecting about 13 million Americans, while more than one million percutaneous transluminal intervention (PCI) procedures are performed annually in the USA. The relative high occurrence of restenosis, despite stent implementation, seems to be the primary limitation of PCI. Over the last decades, single photon emission computed tomography (SPECT) myocardial perfusion imaging (MPI), has proven an invaluable tool for the diagnosis of CAD and patients’ risk stratification, providing useful information regarding the decision about revascularization and is well suited to assess patients after intervention. Information gained from post-intervention MPI is crucial to differentiate patients with angina from those with exo-cardiac chest pain syndromes, to assess peri-intervention myocardial damage, to predict-detect restenosis after PCI, to detect CAD progression in non-revascularized vessels, to evaluate the effects of intervention if required for occupational reasons and to evaluate patients’ long-term prognosis. On the other hand, chest pain and exercise electrocardiography are largely unhelpful in identifying patients at risk after PCI.

Although there are enough published data demonstrating the value of myocardial perfusion SPECT imaging in patients after PCI, there is still debate on whether or not these tests should be performed routinely.

## INTRODUCTION

Coronary artery disease (CAD) is the most prevalent form of cardiovascular disease affecting millions of people all over the world and being a major public health problem. The impact of CAD on the health of individuals in the developed world has declined over the last decades due to the identification of modifiable risk factors and the introduction of new primary and secondary prevention strategies [1]. Revascularization procedures are routinely performed in patients with CAD. However, even with these improvements, the mortality rate is substantially higher than that in the general population. 

Since 1979 when Gruentzing *et al* demonstrated the possibility of using percutaneous transluminal coronary angioplasty (PTCA) in patients with coronary artery disease (CAD), the method has been widely applied worldwide, despite the high restenosis rate of 20-65% in patients without stenting [2-4]. More recently, the introduction of intracoronary stenting has induced a dramatic decline in acute complications and also a significant reduction of the restenosis rate to an average of 20-25% (below 10% with drug-eluting stents’ implementation). As a result, the use of and indications for percutaneous transluminal interventions (PCIs) have greatly expanded [5-9]. 

The development in nuclear myocardial perfusion imaging (MPI) has provided diagnostic and prognostic implications in CAD patients, leading to an explosive increase in the performance of these studies. Specifically, in patients with CAD, MPI provides independent and incremental information in predicting future cardiac events, an essential step in choosing between medical management and revascularization. Variables that predict the likelihood of future events are the extent and severity of inducible ischemia, increased lung uptake of thallium, stress-induced ventricular dilatation and left ventricular ejection fraction (gated-SPECT studies) [10, 11]. 

Chest pain and exercise electrocardiography (ECG) are largely unhelpful in identifying patients at risk after PCI procedures. MPI is of proven value to assess patients post intervention.

MPI is performed either with thallium-201 (^201^Tl) or technetium-99m (^99m^Tc) labeled compounds such as sestamibi and tetrofosmin, while single photon emission computed tomography (SPECT) has replaced planar imaging resulting in significant improvement of MPI scans [10, 11]. Moreover, the use of gated-SPECT provides the possibility to evaluate the left ventricular function parameters [10, 11].

## MPI AND PERCUTANEOUS CORONARY INTERVENTION 

Currently, more than one million PCI procedures are performed annually in the U.S.A. [12]. Nowadays the method is routinely performed mainly in patients with acute myocardial infarction (MI) and unstable coronary syndromes, and is increasingly employed to treat complex lesions, small-diameter vessels, chronic total occlusions, and diseased bypass conduits [1, 2]. In the mid 1990s, the publication of two multicenter trials which demonstrated improved outcomes, led to widespread use of stents [6, 9]. Although increasingly complex lesions and higher-risk patients are being successfully treated percutaneously, restenosis and disease progression continue to cause significant morbidity.

## RESTENOSIS

Restenosis is the major weakness of PCI and remains a main clinical problem while progression of disease at untreated sites occurs at rates approaching 7% per year [1]. Restenosis usually occurs within three to nine months after PCI [3]. PCI restenosis rate without stenting range between 20% and 65%, depending on the method of follow-up and the criteria used to define restenosis [3-9]. Successfully dilated total coronary occlusions have a higher rate of angiographic restenosis at 6 months than dilated stenoses [13, 14]. Coronary angiography is the "gold standard" for detecting restenosis, but its invasive character precludes application in patients following PCI. Chest pain symptoms and non-invasive techniques, such as exercise electrocardiography (ECG) testing, have limited clinical significance for evaluating the efficacy of PCI and detecting restenosis [15].

Patients with symptomatic restenosis develop angina within four to five months after PCI [16]. Angina developing after nine months is usually due to progression of disease at another site [17].

Chest pain following PCI is a poor indicator of restenosis since asymptomatic restenosis occurs in 18% to 59% of patients following PCI and in 30% to 58% of patients after stenting, while up to 45% of patients developing chest pain after PCI do not have angiographic restenosis [16, 18]. Restenosis rate is lower after coronary stenting, ranging to an average of 20-25%, while it is even lower (less than 10%) using drug-eluting stents [9]. On the other hand, current recommendations indicate that individuals with a drug-eluting stent should receive at least 12 months of uninterrupted dual antiplatelet treatment. Patients' assessment should focus on bleeding abnormalities, pre-existing disorders that need anticoagulation treatment, and possible future surgical procedures, since these factors could all contraindicate use of drug-eluting stents [19]. Thus, restenosis remains the Achilles heel of PCI and also a significant clinical problem. 

MPI encounters some difficulties compared to other noninvasive tests; because restenosis is a complex biologic phenomenon evolving over time and the detection of disease by MPI depends on the presence of hemodynamically important stenoses (typically at least 50-70% of the diameter of the lumen), therefore accuracy of stress MPI depends upon the timing after revascularization [20]. When MPI is performed very early after angioplasty or stenting (within 24 - 48 hours), it may exhibit defects in the absence of stenoses. These early defects may be due to abnormal endothelium function which is common after angioplasty and may cause transiently impaired coronary flow reserve after intervention, while the prognostic significance of these defects is uncertain [21]. Some authors found that the incidence of restenosis was 52% to 75% in patients with reversible defects on early (<2 months after PCI) imaging, compared with an incidence of 12% to 17% in those with normal perfusion and thus they stated that MPI performed early after PCI could predict future restenosis [22]. Others noticed that although reversible perfusion defects in the treated vascular territory, within 48 hours of angiographically successful PCI were observed in 54% of patients following PCI or atherectomy, and in 43% of patients after stenting, there was a significant overlap of residual stenosis values between patients with and those without perfusion defects [23]. In conclusion, authors suggested that endothelial dysfunction may be a possible cause of perfusion defect following PCI. The use of stents has lowered the rates of early post-PCI false positive MPI scans and therefore, in cases concerning the adequacy of high-risk angioplasty procedure, early stress MPI scans are considered clinically useful and safe nowadays [24].

On the other hand, ECG gated-SPECT MPI has been used shortly after PCI in patients with acute myocardial infarction, in order to differentiate reversible from persistent left ventricular dysfunction and predict left ventricular functional recovery and late functional outcome. It has been found that greater increase in left ventricular functional recovery occurred in patients with a greater number of segments with perfusion/thickening mismatch early (3 days) after primary PCI, while matched abnormal segments showed no improvement in wall motion score 3 months after PCI and their extent, as was evaluated in the early stage of myocardial infarction, was related to left ventricular ejection fraction at 3 months after PCI [25].

## EVALUATION OF MPI IN DIAGNOSING RESTENOSIS AFTER PCI 

Although there have been many studies reporting high sensitivity and specificity of MPI in detecting restenosis after PCI, nevertheless the literature is limited by verification bias because MPI and angiography - which is considered the “gold standard” evaluation method - were not performed simultaneously but sequentially and results from noninvasive testing probably influenced the decision to perform angiography [26]. In such a study, Hecht and colleagues reported that SPECT thallium testing, when compared to coronary angiography, had a sensitivity and specificity of 96% and 75% in asymptomatic and 99% and 77% in symptomatic patients [27]. However, as previously pointed out, Hecht’s patients did not undergo MPI and coronary angiography simultaneously but sequentially.

In an important meta-analysis of studies published from 1975 to 2000, Garzon and Eisenberg found a pooled sensitivity and specificity of MPI in diagnosing restenosis after PCI, of 87% and 78% respectively [28].

**Fig. (1):**
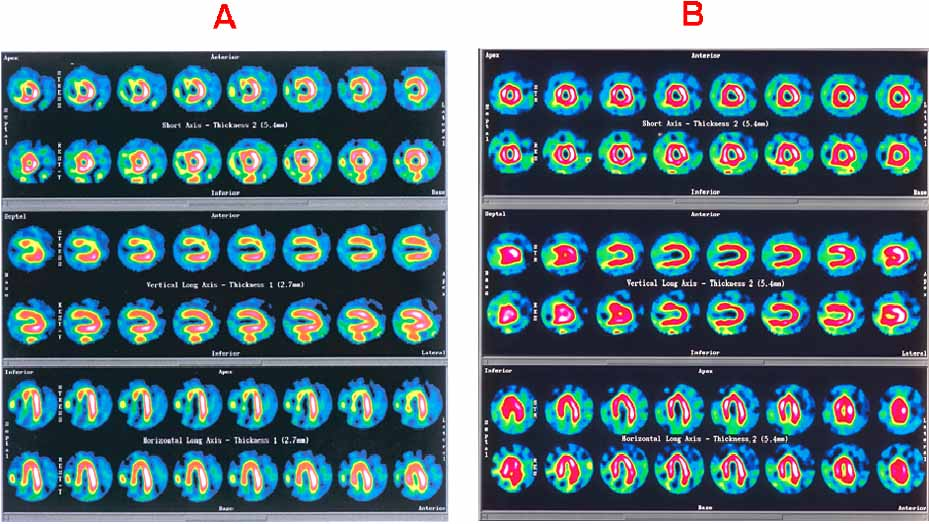
Myocardial perfusion SPECT imaging of a 61-years old patient with one-vessel disease (LAD 95%) before (A) and 6 months after (B) LAD PCI, demonstrated significant improvement in the apex, anterior wall and septum.

In one of our previous studies using Tc-99m tetrofosmin as the radiotracer, sensitivity, specificity, positive and negative predictive value of MPI in detecting restenosis were 81.3%, 88%, 81.3% and 88%, respectively, whereas for the detection of restenosis in specific vessel, the corresponding values were 81.3%, 90%, 76.5% and 89.7%, respectively [29]. In the same study, improvement in late (6 months) post-PCI MPI scans had been observed in most patients with successful PCI confirmed with coronary angiography (Fig. **1**). In some patients, although there was scan improvement, restenosis was found angiographically. Nevertheless, their angiograms revealed less severe stenosis of the corresponding vessels than those taken before PCI. Finally it was suggested that since MPI might be significantly improved irrespective of the presence or absence of restenosis, coronary angiography should be performed only in patients who do not show scan improvement or in other clinically indicated cases [29].

The sensitivity, specificity and accuracy of MPI in predicting restenosis after PCI has also been reported. In a study using dobutamine ^201^Tl MPI, Caner *et al.* found that the method was 76% sensitive, 79% specific, and 77% accurate in predicting restenosis, while the positive and negative predictive values were 66% and 86%, respectively. Sensitivity and specificity related to the vascular territories were: 66-69% for the left anterior descending artery (LAD), 75-100% for the left circumflex artery (LCX) and 83-66% for the right coronary artery (RCA), respectively [30].

MPI for the diagnosis of in stent stenosis (ISS) has also been examined. The mean sensitivity, specificity, positive and negative predictive values and accuracy of MPI were 95%, 73%, 88%, 89%, and 88% respectively [31]. 

Finally, MPI has high sensitivity and specificity in the detection of restenosis after PCI in stenosed coronary grafts. In patients with typical angina the reported sensitivity and specificity are 84% and 80% respectively, while in patients with atypical chest pain or other symptoms the corresponding rates are 70% and 90%, respectively [32].

## MPI AND STRESS ECG

Stress ECG testing, has limited clinical significance for evaluating the efficacy of PCI and detecting restenosis. ECG is much less sensitive and specific in detecting restenosis after PCI compared to MPI, particularly due to the high incidence of baseline electrocardiographic abnormalities in patients with CAD. Also, the usefulness of exercise ECG in patients with partial revascularization is questionable because a positive test may result from non-revascularized areas. Even in patients with dilation of all the stenosed vessels, a positive response may be indicative of disease progression rather than restenosis. Nevertheless, the analysis of other exercise variables such as functional capacity, chronotropic response index, heart-rate recovery and ventricular ectopy during recovery, may improve its diagnostic capacity and provide important prognostic information [33].

Beygui *et al* reported that the sensitivity, specificity and accuracy of stress ECG in asymptomatic patients 6 months after PCI, has been reported to be 53%, 59% and 57% respectively for stress electrocardiography, compared with 63%, 77% and 72% for MPI in the same group of patients [34].

## PROGNOSIS

It seems that the major clinical contribution of MPI in patients post PCI is the significant prognostic value of the method. It has been reported that the detection of ischemia in MPI scans can predict recurrent ischemic events in both symptomatic and asymptomatic patients [35]. The long-term prognostic value of MPI performed late after PCI (i.e., beyond the generally accepted 6-month “restenosis window”) has been evaluated in several studies. These studies showed that the occurrence of cardiac events (death, nonfatal infarction, and late revascularization procedures) was higher in the presence of myocardial ischemia in the MPI, in both symptomatic and asymptomatic patients, while the event-free survival curves showed a higher event rate in patients with than those without ischemia. The summed stress score (SSS) and summed difference score (SDS) were independent significant predictors of cardiac events [15, 36-38]. 

Specifically, Cottin *et al*., studied 152 patients 5 ± 2 months after stenting with stress-rest thallium SPECT and reported a significant worse prognosis of ischemic patients compared with non-ischemic (58% had major cardiac events or revascularization vs 11%, p< 0.001) [39]. In addition, Ho *et al*., studying the late outcome of a series of 211 patients who underwent a SPECT ^201^Tl MPI 1-3 years after PTCA, reported that the SSS index was significantly associated with the end point of cardiac death or myocardial infraction [40]. Similarly, Acampa *et al*., in a study population of 206 patients who underwent a ^99m^Tc-MIBI myocardial SPECT 12-18 months post PCI, found that SSS and SDS were significant predictors of cardiac events, while the occurrence of cardiac events was higher in the presence of ischemia at SPECT in symptomatic and symptom-free patients (both p<0.001) [36]. In a larger study, Zellweger *et al*. evaluated 356 patients (both symptomatic and asymptomatic) with a ^99m^Tc-MIBI myocardial SPECT 6 months after stenting and follow-up was an average of about 4 years. A higher degree of ischemia measured by SDS was associated with a higher rate of death, myocardial infarction and repeat revascularization, while SPECT imaging added incremental information for the prediction of critical events [41]. Additionally, similar results have been reported by Solodky *et al*. recently [42]. Comparably, Zhang *et al* studying 318 patients who underwent a ^99m^Tc-MIBI SPECT 10±12 months after PCI, reported that SSS was the best independent predictor for hard cardiac events (cardiac death or myo-cardial infarction) and SDS was the best independent predictor for late revascularization (both p<0.001) [15]. Recently, Galassi *et al*., studied 322 consecutive patients with ^99m^Tc-tetrofosmin after incomplete revascularization and found that nuclear data provided significant incremental prognostic value for cardiac events compared with the clinical, exercise testing and angiographic findings (p<0.01) [43]. 

In two recently published studies, we evaluated the long-term prognostic value of ^99m^Tc-tetrofosmin myocardial gated-SPECT, in asymptomatic patients after coronary artery stenting [37, 38]. We included 246 consecutive patients in the study, who underwent exercise gated-SPECT myocardial imaging 5-7 months after PCI and were followed-up for a mean period of 8.3 years (SD=2.9). Cardiovascular death and non-fatal myocardial infarction were considered as hard cardiac events, while late revascularization procedures as soft events. When multiple Cox regression analysis was implied, the factors which remained significant in the final model for soft events were SSS, SDS, and angina during exercise testing [37]. In addition, SSS, SDS and left ventricular dilation were independently associated with hard cardiac events as defined from the results of multiple analysis [37]. However, SSS and SDS were the only independent predictors for both hard and soft events. Moreover, ^99m^Tc-tetrofosmin myocardial gated-SPECT, provided incremental prognostic information, over clinical and exercise test data, for the prediction of cardiac events in asymptomatic patients after PCI [38].

However, in these studies there is a general agreement that chest pain symptoms are of limited value to predict future cardiac events [15, 36-38, 42]. In addition, Elhendy *et al*. studying 381 patients 4.5 ± 3.2 years after myocardial revascularization (CABG in 201 patients, PCI in 180 patients) with ^99m^Tc-tetrofosmin, found a similar incidence of hard cardiac events (cardiac death, non fatal myocardial infraction) in patients with and without angina before MPI [44]. In fact, they submitted that symptoms were not predictive of outcome, while an abnormal MPI indicative of myocardial ischemia, was independently associated with the composite endpoints of hard cardiac events and late revascularization, adding significant incremental prognostic value to clinical data in the prediction of hard cardiac events (p<0.01) [44]. On the contrary, a normal study identifies a very low-risk population (no hard cardiac events during follow-up), irrespectively of the method of previous revascularization, in whom no further intervention is required. A conclusion of the above studies was that the absence of symptoms should not be interpreted as an indicator of a low-risk status, therefore asymptomatic patients should not be deferred from MPI stress testing [37, 38, 44].

## RECOMMENDATIONS FOR MPI

Routine stress MPI is not recommended yet in all patients after PCI in order to predict cardiac events and especially in patients after coronary stenting where the application of drug-eluting stents and the use of dual antiplatelet therapy have improved patients prognosis [33]. In addition, given the high false-positive rate of MPI early (<3 months) after PCI, patients who develop chest pain within this time should undergo angiography, as well as patients who present typical angina, ECG changes or elevated cardiac enzymes 3 to 6 months after PCI [1, 33]. Patients lacking such characteristics, who develop atypical symptoms, should undergo MPI. Although the specificity of MPI 3 to 6 months following PCI might be limited, a normal study should reliably exclude restenosis. If perfusion is abnormal, angiography should be considered as the next step. The “ACCF / ASNC / ACR / AHA / ASE / SCCT / SCMR / SNM Appropriate Use Criteria for Cardiac Radionuclide Imaging” (2009), has recommended as “inappropriate” the general application of SPECT MPI in the first 2 years post PCI to evaluate asymptomatic patients, although such a follow-up is recommended as “appropriate” for asymptomatic patients after incomplete revascularization, whereas an additional revascularization seems feasible [45]. On the other hand, since the accuracy and prognostic value of MPI performed 6 or more months after PCI is significant, it could be recommended that asymptomatic patients and particularly those who were also asymptomatic prior the intervention, should initially be followed clinically and undergo MPI less than 2 years (probably 6 to 12 months) after PCI, for further risk stratification [1, 15, 36 - 39, 41, 44]. Patients with normal and low- or intermediate-risk scans (small or medium-sized defects of mild-to-moderate severity) can be managed conservatively. Patients with high-risk scans (medium-sized severe defects, large defects of any severity, or scans showing stress-induced left ventricular failure) should undergo angiography. Thereafter, asymptomatic patients should undergo MPI every 1 to 3 years unless symptoms develop, where MPI should be performed sooner [1, 41].

MPI is generally indicated in high risk patients who have worse long-term prognosis compared to other patients, such as diabetic patients in whom silent ischemia occurs commonly after angioplasty, patients with heart failure who experience major adverse cardiac events more frequently, and patients with saphenous vein graft interventions [32, 35, 46-48]. The occurrence of ischemia in MPI of asymptomatic diabetic patients was found to be associated with a high risk for repeat interventional procedures although no difference in major cardiac events was noticed compared to symptomatic diabetic patients [48].

## CONCLUSION

Over the last decades, SPECT MPI has proven an invaluable tool for evaluating patients in cardiovascular medicine. In addition, PCI procedures are widely used in patients with CAD. By assessing myocardial perfusion, SPECT imaging aids in diagnosis of CAD and patient risk stratification, providing important information on extent of myocardium at risk and scar- myocardial viability, disease progression, hemodynamic significance of coronary artery stenoses (culprit lesions) and myocardial function using gated-SPECT technique. Evaluating the aforementioned data, nuclear imaging helps in decision about revascularization and is well suited to assess patients after intervention. The literature has demonstrated the usefulness of nuclear imaging after PCI, providing significant data in patients with recurrent symptoms after revascularization, and more importantly, providing prognostic information after the intervention, independently of symptoms. Chest pain and exercise ECG could be considered unhelpful in identifying patients at risk after revascularization but MPI is of proven value, although there is still debate on whether or not myocardial perfusion SPECT should be performed routinely and also about the certain time period. As PCI techniques expand and evolve, mainly with the increasing use of drug-eluting stents with low restenosis rate, the role of nuclear imaging will require further investigation.

## References

[1] Giedd KN, Bergmann SR (2004). Myocardial perfusion imaging following percutaneous coronary intervention. The importance of restenosis, disease progression, and directed reintervention. J Am Coll Cardiol.

[2] Gruentzig AR, Senning A, Siegethaler WE (1979). Nonoperative dilation of coronary artery stenosis: Perqutaneous transluminal coronary angioplasty. N Engl J Med.

[3] Callif RM, Fortin DF, Frid DJ (1991). Restenosis after coronary angioplasty: an overview. J Am Coll Cardiol.

[4] Gruentzig AR, King SB, Schlumpf M, Siegenthaler W (1987). Long-term follow-up after perqutaneous transluminal coronary angioplasty: the early Zurich experience. N Engl J Med.

[5] Goy JJ, Eeckhout E (1998). Intracoronary stenting. Lancet.

[6] Serruys P, de Jaegere P, Kiemeneij F (1994). A comparison of balloon-expandable-stent implantation with balloon angioplasty in patients with coronary artery disease. N Engl J Med.

[7] Kastrati A, Schömig A, Elezi S (1999). Prognostic value of the modified American College of Cardiology / American Heart Association stenosis morphology classification for long-term angiographic and clinical outcome after coronary stent placement. Circulation.

[8] De Jaegere PP, Eefting FD, Popma JJ, Seruys PW (1996). Clinical trials on intracoronary stenting. Semin Interv Cardiol.

[9] Dehmer GJ, Smith KJ (2009). Drug-eluting coronary artery stents. Am Fam Physician.

[10] Gershlick AH, de Belder M, Chambers J GI (2007). Role of non-invasive imaging in the management of coronary artery disease: an assessment of likely change over the next 10 years. A report from the British Cardiovascular Society Working Group. Heart.

[11] Underwood SR, Anagnostopoulos C, Cerqueira M (2004). Myocardial scintigraphy: the evidence. Eur J Nucl Med Mol Imaging.

[12] Adams GL, Ambati SR, Adams JM, Borges-Neto S (2006). Role of nuclear imaging after coronary revascularization. J Nucl Cardiol.

[13] Pfisterer ME, Zellweger MJ, Ell PJ, Gambhir SS (2004). Nuclear medicine in clinical diagnosis and treatment.

[14] Violaris AG, Melkert R, Serruys PW (1995). Long-term luminal renarrowing after successful elective coronary angioplasty of total occlusions. A quantitative angiographic analysis. Circulation.

[15] Zhang X, Liu X, He ZX (2004). Long-term prognostic value of exercise 99mTc-MIBI SPET myocardial perfusion imaging in patients after perqutaneous coronary intervention. Eur J Nucl Med Mol Imaging.

[16] Holmes DR, Vlietstra RE, Smith HC (1984). Restenosis after percutaneous transluminal coronary angioplasty (PTCA): a report from the PTCA Registry of the National Heart, Lung, and Blood Institute. Am J Cardiol.

[17] Suresh CG, Grant SC, Henderson RA, Bennett DH (1993). Late symptom recurrence after successful coronary angioplasty: angiographic outcome. Int J Cardiol.

[18] Ruygrok PN, Webster MW, de Valk V (2001). Clinical and angiographic factors associated with asymptomatic restenosis after percutaneous coronary intervention. Circulation.

[19] Bavry AA, Bhatt DL (2008). Appropriate use of drug-eluting stents: balancing the reduction in restenosis with the concern of late thrombosis. Lancet.

[20] Mintz GS (2000). Remodeling and Restenosis: Observations from Serial Intravascular Ultrasound Studies. Curr Interv Cardiol Rep.

[21] Jaffe R, Haim SB, Karkabi B (2002). Myocardial perfusion abnormalities early (12-24 h) after coronary stenting or balloon angioplasty: Implications regarding pathophysiology and late clinical outcome. Cardiology.

[22] Rodes-Cabau J, Candell-Riera J, Domingo E (2001). Frequency and clinical significance of myocardial ischemia detected early after coronary stent implantation. J Nucl Med.

[23] Bachmann R, Sechtem U, Voth E, Schroder J, Hopp HW, Schicha H (1997). Dipyridamole scintigraphy and intravascular ultrasound after successful coronary intervention. J Nucl Med.

[24] Kim DW, Park SA, Kim CG, Lee C, Oh SK, Jeong JW (2008). Reversible defects on myocardial perfusion imaging early after coronary stent implantation: a predictor of late restenosis. Int J Cardiovasc Imaging.

[25] Kurihara H, Nakamura S, Takehana K (2005). Scintigraphic prediction of left ventricular functional recovery early after primary coronary angioplasty using single-injection quantitative electrocardiographic gated SPECT. Nucl Med Commun.

[26] Lauer MS (2000). Role of stress testing and cardiac imaging in patients who have undergone previous coronary revascularization. Cardiol Rev.

[27] Hecht HS, Shaw RE, Chin HL (1991). Silent ischemia after coronary angioplasty: Evaluation of restenosis and extent of ischemia in asymptomatic patients by tomographic thallium-201 exercise imaging and comparison with symptomatic patients. J Am Coll Cardiol.

[28] Garzon PP, Eisenberg MJ (2001). Functional testing for the detection of restenosis after percutaneous transluminal coronary angioplasty: a meta-analysis. Can J Cardiol.

[29] Georgoulias P, Demakopoulos N, Kontos A (1998). Tc-99m tetrofosmin myocardial perfusion imaging before and six months after percutaneous transluminal coronary angioplasty. Clin Nucl Med.

[30] Caner B, Oto A, Ovunc K, Kiratli P (1998). Prediction of restenosis after successful percutaneous coronary angioplasty by dobutamine thallium-201 scintigraphy. Int J Cardiol.

[31] Milavetz JJ, Miller JD, Hodge DO, Holmes DR, Gibbons RJ (1998). Accuracy of single-photon emission computed tomography myocardial perfusion imaging in patients with stents in native coronary arteries. Am J Cardiol.

[32] Lakkis NM, Mahmarian JJ, Verani MS (1995). Exercise thallium-201 single photon emission computed tomography for the evaluation of coronary artery bypass graft patency. Am J Cardiol.

[33] Rajagopal V, Lauer MS, Zaret BL, Beller GA (2005). Clinical nuclear cardiology: state of the art and future directions.

[34] Beygui F, Le Feuvre C, Maunoury C (2000). Detection of coronary restenosis by exercise electrocardiography thallium-201 perfusion imaging and coronary angiography in asymptomatic patients after percutaneous transluminal coronary angioplasty. Am J Cardiol.

[35] Pfisterer M, Rickenbacher P, Kiowski W, Muller-Brand J, Burkart E (1993). Silent ischemia after percutaneous transluminal coronary angioplasty: incidence and prognostic significance. J Am Coll Cardiol.

[36] Acampa W, Petretta M, Florimonte L, Mattera A, Cuocolo A (2003). Prognostic value of exercise cardiac tomography performed late after percutaneous coronary intervention in symptomatic and symptom-free patients. Am J Cardiol.

[37] Georgoulias P, Demakopoulos N, Tzavara C (2008). Long-term prognostic value of Tc-99m tetrofosmin myocardial gated-SPECT imaging in asymptomatic patients after percutaneous coronary intervention. Clin Nucl Med.

[38] Georgoulias P, Tzavara C, Demakopoulos N (2008). Incremental prognostic value of ^99m^Tc-tetrofosmin myocardial SPECT after percutaneous coronary intervention. Ann Nucl Med.

[39] Cottin Y, Rezaizadeh K, Touzery C (2001). Long-term prognostic value of 201Tl single-photon emission computed tomographic myocardial perfusion imaging after coronary stenting. Am Heart J.

[40] Ho KT, Miller TD, Holmes DR, Hodge DO, Gibbons RJ (1999). Long-term prognostic value of Duke treadmill score and exercise thallium-201 imaging performed one to three years after percutaneous transluminal coronary angioplasty. Am J Cardiol.

[41] Zellweger MJ, Weinbacher M, Zutter AW (2003). Long-term outcome of patients with silent versus symptomatic ischaemia six months after percutaneous coronary intervention and stenting. J Am Coll Cardiol.

[42] Solodky A, Assali AR, Mats I (2007). Prognostic value of myocardial perfusion imaging in symptomatic and asymptomatic patients after percutaneous coronary intervention. Cardiology.

[43] Galassi AR, Grasso C, Azzarelli S, Ussia G, Moshiri S, Tamburino C (2006). Usefulness of exercise myocardial scintigraphy in multivessel coronary disease after incomplete revascularization with coronary stenting. Am J Cardiol.

[44] Elhendy A, Schinkel AF, van Domburg RT, Bax JJ, Valkema R, Poldermans D (2003). Risk stratification of patients after myocardial revascularization by stress Tc-99m tetrofosmin myocardial perfusion tomography. J Nucl Cardiol.

[45] Hendel RC, Berman DS, Di Carli MF, ACCF/ASNC/ ACR/AHA/ASE/SCCT/SCMR/SNM 2009 Appropriate Use Criteria for Cardiac Radionuclide Imaging: A Report of the American College of Cardiology Foundation Appropriate Use Criteria Task Force, the American Society of Nuclear Cardiology, the American College of Radiology, the American Heart Association, the American Society of Echocardiography, the Society of Cardiovascular Computed Tomography, the Society for Cardiovascular Magnetic Resonance, and the Society of Nuclear Medicine (2009). Endorsed by the American College of Emergency Physicians. J Am Coll Cardiol.

[46] Anderson RD, Ohman EM, Holmes DR (1998). Prognostic value of congestive heart failure history in patients undergoing percutaneous coronary interventions. J Am Coll Cardiol.

[47] Keeley EC, Velez CA, O’Neill WW, Safian RD (2001). Long-term clinical outcome and predictors of major adverse cardiac events after percutaneous interventions on saphenous vein grafts. J Am Coll Cardiol.

[48] L’Huillier I, Cottin Y, Touzery C (2003). Predictive value of myocardial tomoscintigraphy in asymptomatic diabetic patients after percutaneous coronary intervention. Int J Cardiol.

